# Reducing the global burden of musculoskeletal conditions

**DOI:** 10.2471/BLT.17.204891

**Published:** 2018-04-12

**Authors:** Andrew M Briggs, Anthony D Woolf, Karsten Dreinhöfer, Nicole Homb, Damian G Hoy, Deborah Kopansky-Giles, Kristina Åkesson, Lyn March

**Affiliations:** a School of Physiotherapy and Exercise Science, Curtin University, GPO Box U1987, Perth, 6845, Australia.; b Bone and Joint Research Office, Royal Cornwall Hospital, Cornwall, England.; c Department for Musculoskeletal Rehabilitation, Prevention and Health Service Research, Charité Universitätsmedizin, Berlin, Germany.; d Department of Essential Medicines and Health Products, World Health Organization, Geneva, Switzerland.; e Sydney Medical School, The University of Sydney, Sydney, Australia.; f Canadian Memorial Chiropractic College, University of Toronto, Toronto, Canada.; g Department of Clinical Sciences, Lund University, Malmö, Sweden.; h Institute of Bone and Joint Research, Royal North Shore Hospital, Sydney, Australia.

## Importance of musculoskeletal health

Musculoskeletal conditions include more than 150 diagnoses that affect the locomotor system. These conditions are characterized by pain and reduced physical function, often leading to significant mental health decline, increased risk of developing other chronic health conditions and increased all-cause mortality.^1^ Many musculoskeletal conditions share risk factors common to other chronic health conditions, such as obesity, poor nutrition and a sedentary lifestyle. Musculoskeletal conditions account for the greatest proportion of persistent pain across geographies and ages.^2^ Back and neck pain, osteoarthritis, rheumatoid arthritis and fractures are among the most disabling musculoskeletal conditions and pose major threats to healthy ageing by limiting physical and mental capacities and functional ability. Although the prevalence of major musculoskeletal conditions increases with age, they are not just conditions of older age. Regional pain conditions, low back and neck pain, musculoskeletal injury sequelae and inflammatory arthritides commonly affect children, adolescents and middle-aged people during their formative and peak income-earning years, establishing trajectories of decline in intrinsic capacity in later years. While point prevalence estimates vary with respect to age and musculoskeletal condition, approximately one in three people worldwide live with a chronic, painful musculoskeletal condition. Notably, recent data suggest that one in two adult Americans live with a musculoskeletal condition, a prevalence comparable to that of cardiovascular and chronic respiratory diseases combined, which cost 213 billion United States dollars in 2011 (or 1.4% of gross domestic product).^3^ Data from low- and middle-income countries are fewer, yet comparable.^4^

Musculoskeletal health is critical for human function, enabling mobility, dexterity and the ability to work and actively participate in all aspects of life. Musculoskeletal health is therefore essential for maintaining economic, social and functional independence, as well as human capital across the life course. Impaired musculoskeletal health is responsible for the greatest loss of productive life years in the workforce compared with other noncommunicable diseases,^5^ commonly resulting in early retirement and reduced financial security. In subsistence communities and low- and middle-income economies, impaired musculoskeletal health has profound consequences on an individual’s ability to participate in social roles and in the prosperity of communities.^4^

## Burden of disease

Burden of disease profiles are shifting from communicable, neonatal, maternal and nutritional health conditions to predominantly long-term noncommunicable diseases, commonly including musculoskeletal conditions. For example, noncommunicable diseases accounted for 61.4% of global disability-adjusted life years (DALYs) in 2016, compared to 43.9% in 1990.^6^ The steepest trajectory of rise in the burden of such diseases was observed in low-income settings.^6^ With this transition in health profiles, the global population is now living longer with consequences of chronic disease and injuries, particularly musculoskeletal conditions. This demographic shift underlines the importance of re-focusing the emphasis of health care from curative to promotive, preventive and rehabilitative health care,^6^^–^^8^ particularly in low- and middle-income settings. This is also relevant in high-income settings, where over-medicalization and an emphasis on a biomedical, rather than biopsychosocial approach to care, can lead to poor or adverse health outcomes and unsustainable health care expenditure. The opioid medicine epidemic for management of non-cancer pain, the majority of which is of musculoskeletal etiology, is a notable example. Prioritizing community and primary health-care services and a long-term care system will have the greatest impact on improving functional ability into older age and containing health care expenditure.^7^

The 2016 Global Burden of Disease (GBD) data for noncommunicable diseases identified the profound burden of disease associated with musculoskeletal health. DALYs for musculoskeletal conditions increased by 61.6% between 1990 and 2016, with an increase of 19.6% between 2006 and 2016. Osteoarthritis was observed to have a 104.9% rise in DALYs (or 8.8% when age-standardized) from 1990 to 2016.^6^ Musculoskeletal conditions comprised the second highest global volume of years lived with disability in 2016.^8^ Spinal pain remains the leading cause of global disability since 1990. Notably, these GBD estimates likely underestimate the true burden of musculoskeletal health conditions since important constructs such as carer burden, participation and financial implications are not considered.^9^

## The need for integrated care

More than half of all older people experience multimorbidity of noncommunicable diseases. Such multimorbidities increase with age and are more common among those in lower socioeconomic groups.^10^ This reinforces the need to address noncommunicable diseases in a whole-person, integrated manner rather than with an approach where individual conditions are managed in silos.^7^ Multimorbidity very commonly includes musculoskeletal conditions, with musculoskeletal prevalence ranging from one-third to more than one-half of all noncommunicable disease multimorbidity presentations.^11^ Importantly, the presence of a musculoskeletal condition significantly depletes physical function, clusters with mental health impairment and increases health-care costs.^11^ These data highlight that policies, strategies and health programmes for noncommunicable diseases, as well as essential care packages for universal health coverage (UHC), must include musculoskeletal health as an integral component, particularly those programmes targeted in lower socioeconomic settings and for older people.

## Opportunity for action

The sustainable development goals (SDGs) and the Decade of Healthy Ageing 2020–2030^12^ offer a timely and favourable opportunity for increased global attention and action on musculoskeletal health. To achieve the *2030 agenda for sustainable development* and to promote and maintain health across the life course, a renewed and sustained focus on improving musculoskeletal health is needed at national and global levels. While the Bone and Joint Decade 2000–2010^13^ catalysed awareness of the burden of musculoskeletal health conditions, important gaps in health system improvements remain and a significant proportion of the global population continues to live with disabling musculoskeletal conditions, irrespective of age, race and geography.^1^

Three priorities for action to reduce the global disability burden exist. First, there are substantial opportunities for global leadership to support policy responses which have so far been neglected. For example, the *2008–2013 Action plan for the global strategy for the prevention and control of noncommunicable diseases* focused on mortality associated with cardiovascular disease, cancer, diabetes and chronic respiratory disease, rather than on strategies to promote living with improved intrinsic capacity. While the nine global targets within the *Global action plan for the prevention and control of noncommunicable diseases 2013–2020*^14^ are relevant to the prevention and management of musculoskeletal health conditions, musculoskeletal health is not identified as a priority area for noncommunicable disease management and important occupational and environmental targets are not considered.^15^ Musculoskeletal health was only included as a noncommunicable disease target since 2016 in the *Action plan for the prevention and control of noncommunicable diseases in the WHO European Region*.^16^ The World Health Organization and its Member States can help reduce the global disability burden through an increased focus on musculoskeletal health within system-reform initiatives for noncommunicable diseases and healthy ageing policy agendas. There is a wealth of evidence for what works to improve musculoskeletal health outcomes,^17^ yet translation into policy and practice remains limited.^18^ Explicit advocacy for, and integration of, musculoskeletal health and persistent pain into existing global and/or regional policy reform initiatives will be important to drive appropriate policy and service implementation, particularly as part of action towards the SDGs.

Second, targets and monitoring for functional ability should be set as part of noncommunicable diseases global health surveillance and as part of the health SDG performance targets. SDG 3 aims to ensure healthy lives and promote wellbeing for all at all ages, which implies support for functional independence and participation. However, the specific target for noncommunicable diseases remains focused on reducing premature mortality from such diseases by one-third by 2030. This target is critical because premature mortality from such diseases disproportionally affects people in low- and middle-income countries, the poorest and most vulnerable; however, targets to reduce disability related to noncommunicable diseases, as the major contributor to global DALYs, are absent.^6^^,^^8^ While musculoskeletal health conditions may be indirectly addressed as part of the SDG on health, particularly in the context of preventive actions that influence comorbidities such as obesity, current performance targets would not reflect changes in musculoskeletal-related disability. Global targets should also be set to reflect maintenance of mobility, participation and physical function as key components of functional ability and performance.

Third, musculoskeletal health should be part of noncommunicable diseases national policy reform. National system-level health policy and strategy responses to address musculoskeletal health as a component of noncommunicable diseases care remain disproportionate with the burden of disease.^1^ While health systems are now responding to the burden of noncommunicable diseases, there has been an almost exclusive focus on cancer, diabetes, chronic respiratory disease and cardiovascular disease and, more recently, mental health. While these foci are important, inadequate prioritization of musculoskeletal health and persistent pain as part of health reform initiatives targeting noncommunicable diseases does not align with contemporary evidence for global health, limiting opportunities for development of appropriate integrated policy responses, workforce capacity building initiatives and harnessing of capacity in civil society. System reform leadership in some high-, middle- and low-income regions is nonetheless encouraging.^18^ For example, the development of person-centred models of care for musculoskeletal health and persistent pain that consider multimorbidity and care integration across the health and social care systems are recognized to improve policy capacity, service delivery and cost–effectiveness.^1^^,^^18^ Implementation strategies have been developed for high-, middle- and low-income settings. A global framework to develop, implement and evaluate such models has also been established.^18^ Further development and dissemination of effective models of care is needed to inform promotive, preventive, rehabilitative and curative essential packages for UHC; innovative service delivery options; and strategies to build workforce capacity and consumers’ capacity to actively participate in care.

Service- and system-level responses addressing musculoskeletal health should also integrate the responses to other noncommunicable diseases. This will have the greatest impact if organizations that focus on noncommunicable diseases and injury work cooperatively to tackle the crosscutting challenges of health system reform.
